# Supplementary material for: Unchanged frequency and decreasing magnitude of outbursts from ice-dammed lakes in Alaska
Source: Nat Commun. 2023 Oct 2;14:6138. doi: 10.1038/s41467-023-41794-6 (PMC10545694; doi:10.1038/s41467-023-41794-6)
Supplement: Supplementary file 1 — Supplementary Information [file 41467_2023_41794_MOESM1_ESM.pdf]

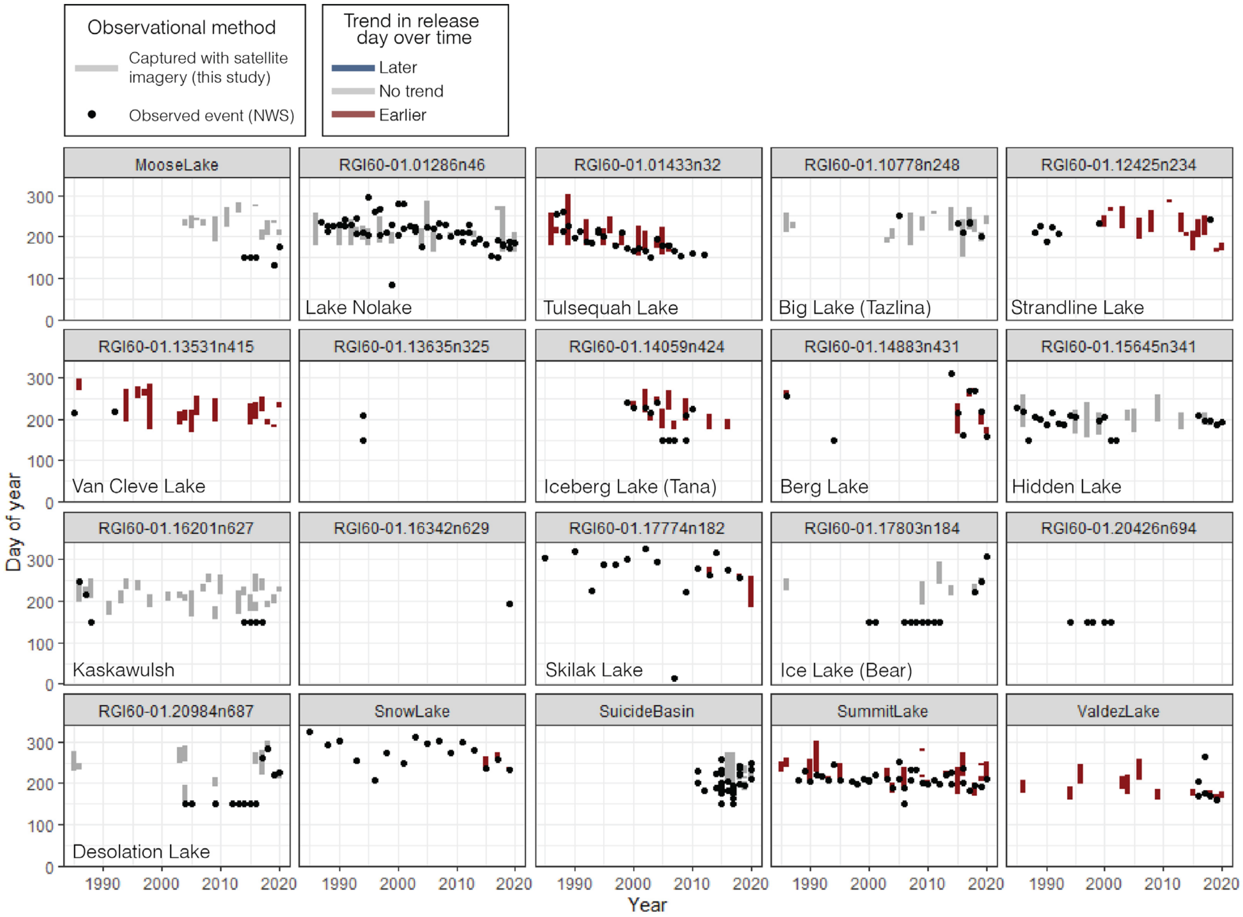

Fig. S1 | Comparison between events documented in this study and previously recorded events. Segments signify the last date a lake was full to the first day it was drained identified in satellite imagery, and black dots represent the observed lake release date previously compiled by Veh et al.[1], including events from the Alaska Glacier Dammed Lake dataset[2]. If an event had only a year and no date specified, the event is plotted at day 150.

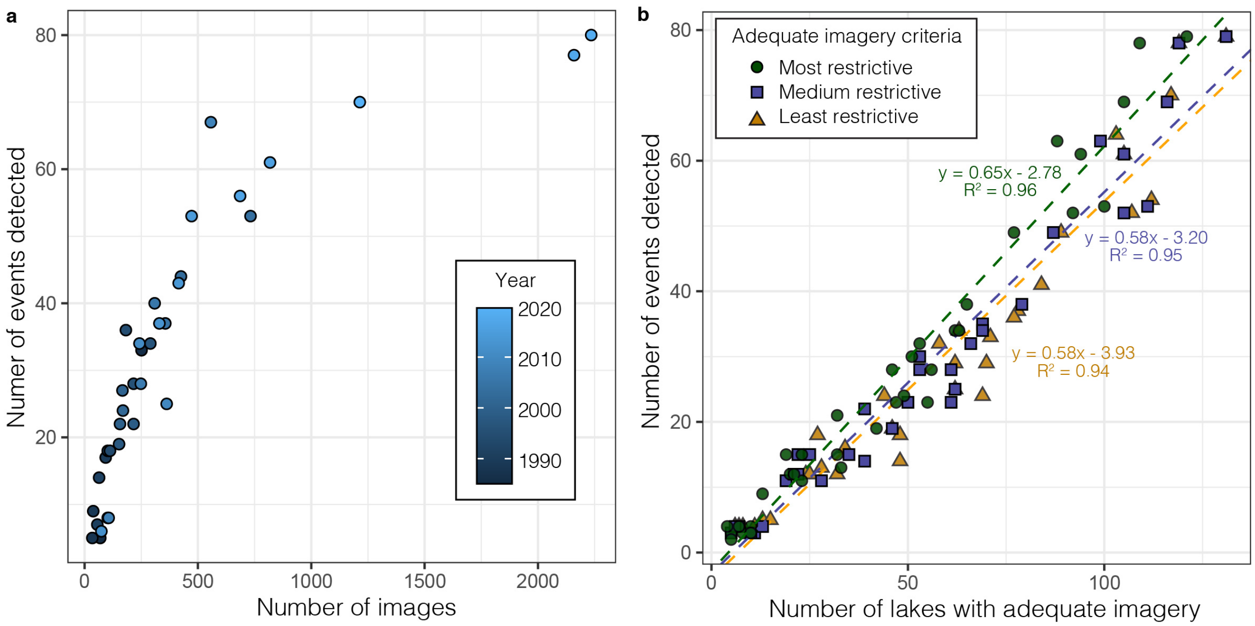

Fig. S2 | Correlation between the number of images available each year, number of lakes with adequate imagery, and the number of drainage events detected. a, Relationship between the total number of images available and number of drainage events detected colored by year, and **b,** relationship between the number of events detected and the number of lakes with adequate imagery for the three different criteria (see Fig. S3).

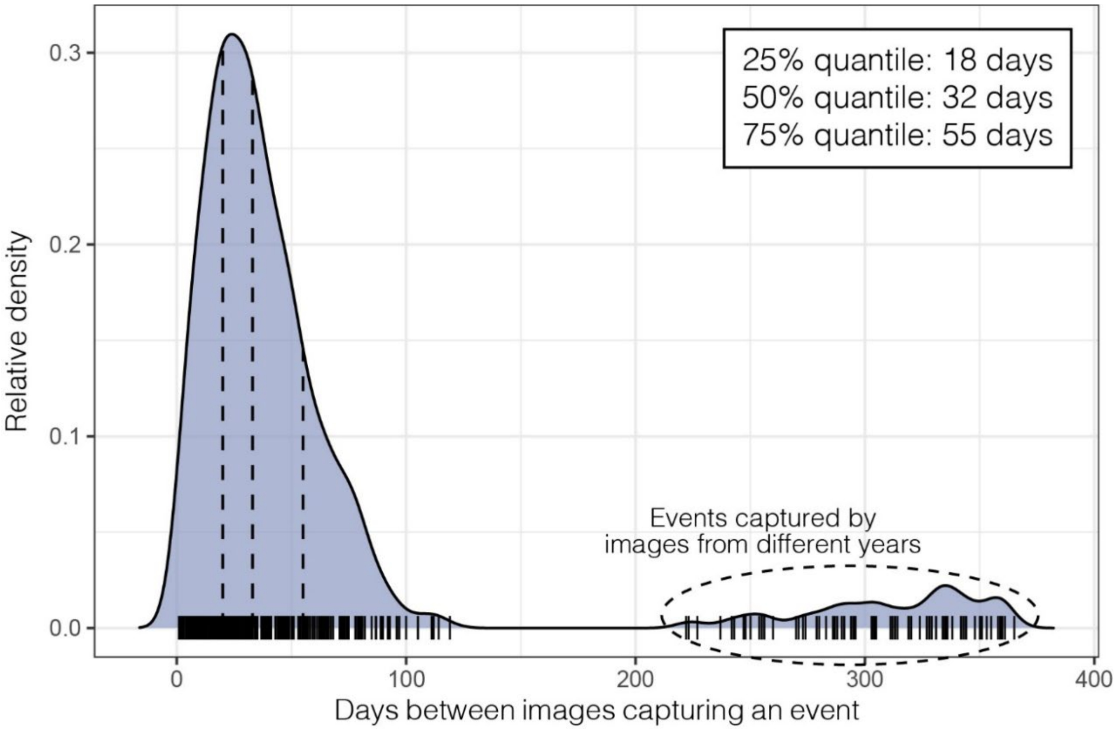

Fig. S3 | Empirical probability density function of number of days between images which captured a lake drainage event. Black ticks represent an individual event, and dashed lines represent the 25%, 50%, and 75% quantiles.

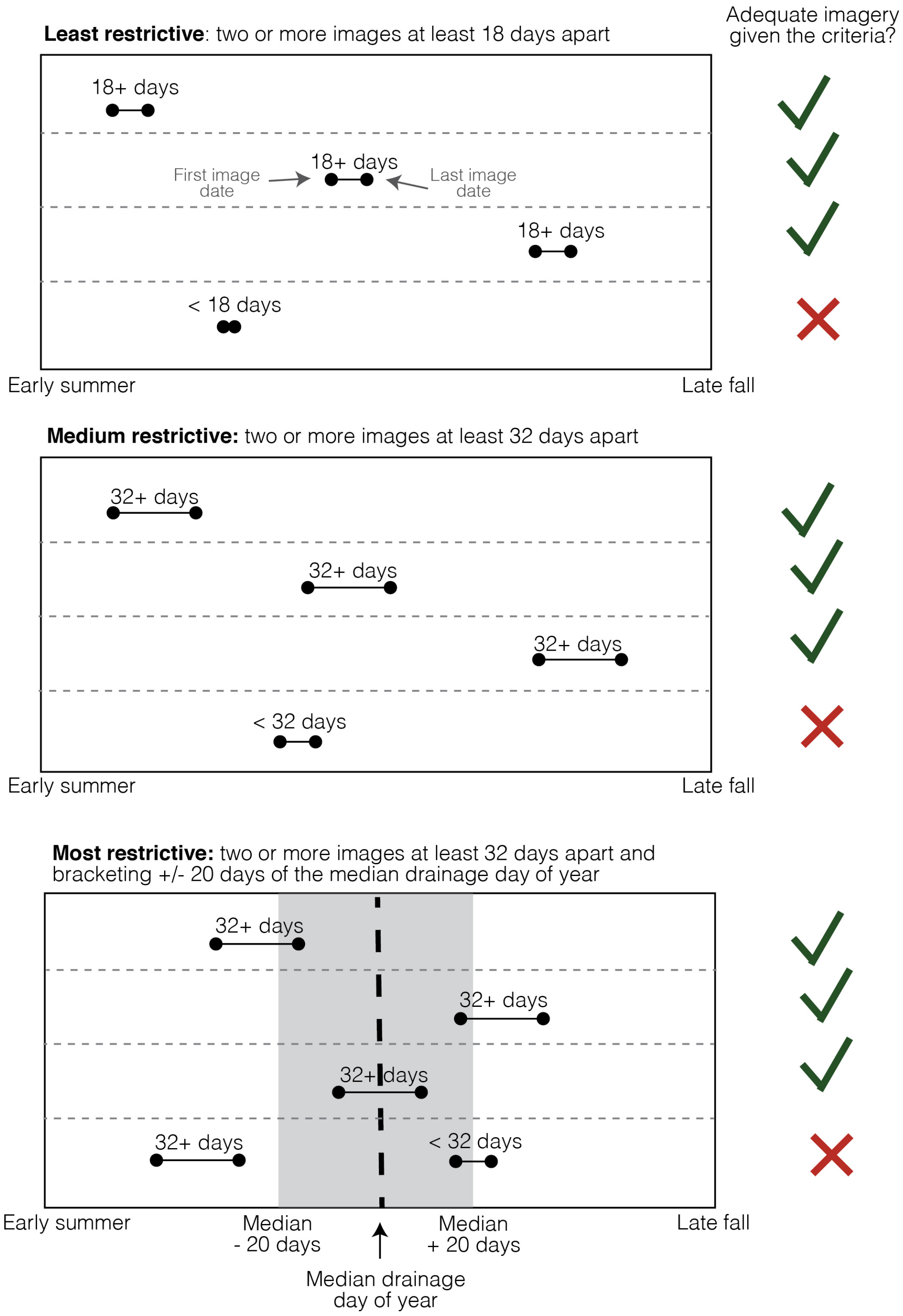

Fig. S4 | Visual representation of the three different criteria used to define whether a lake had adequate imagery to detect an event in any given year. The three criteria includes the least restrictive criteria (at least two images that are 18 or more days apart), the medium restrictive criteria (at least two images that are 32 or more days apart), and the most restrictive (two images at least 32 or more days apart, taking the timing of the images into consideration).

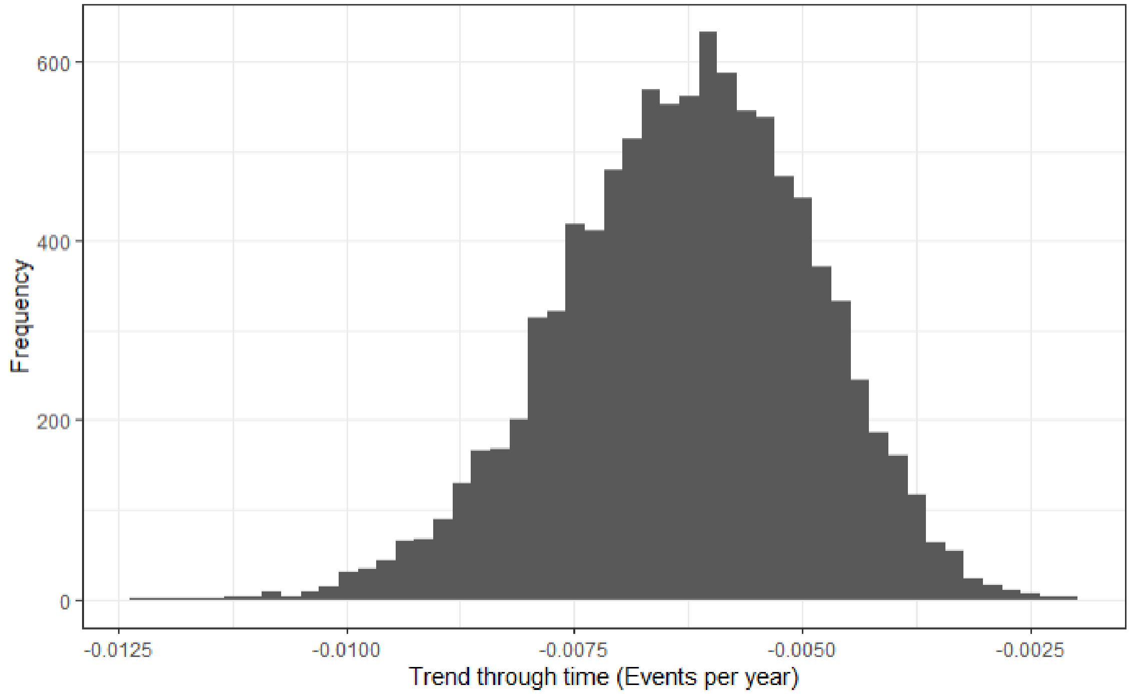

Fig. S5 | Distribution of trends over time from bootstrapping. Results of sampling 50 images per year and calculating the trend in frequency of events over time 10,000 times. All instances resulted in near zero slopes (x-axis), showing no trend in time once image availability is held constant.

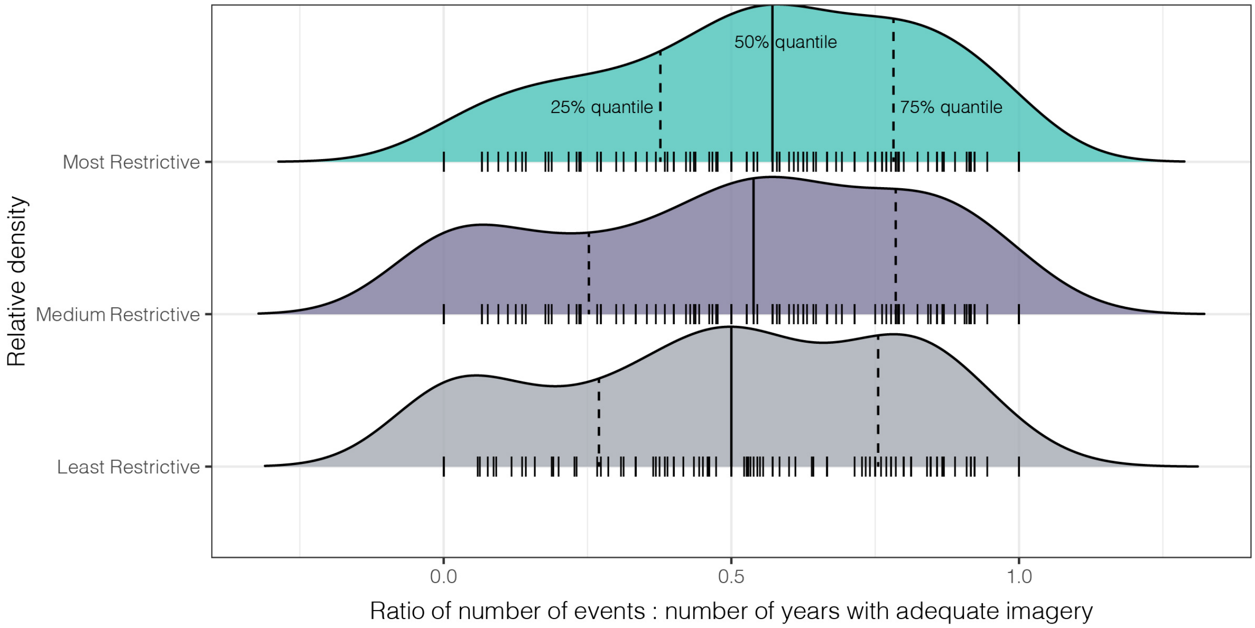

Fig. S6 | Smoothed density distribution of the ratio of number of events to the number of years with adequate imagery given the three different criteria (see Fig. S3). Black ticks represent individual lakes and vertical lines represent the 25%, 50%, and 75% quantiles. A value of 0.5 indicates that a lake drained 50% of all years where adequate imagery was detected.

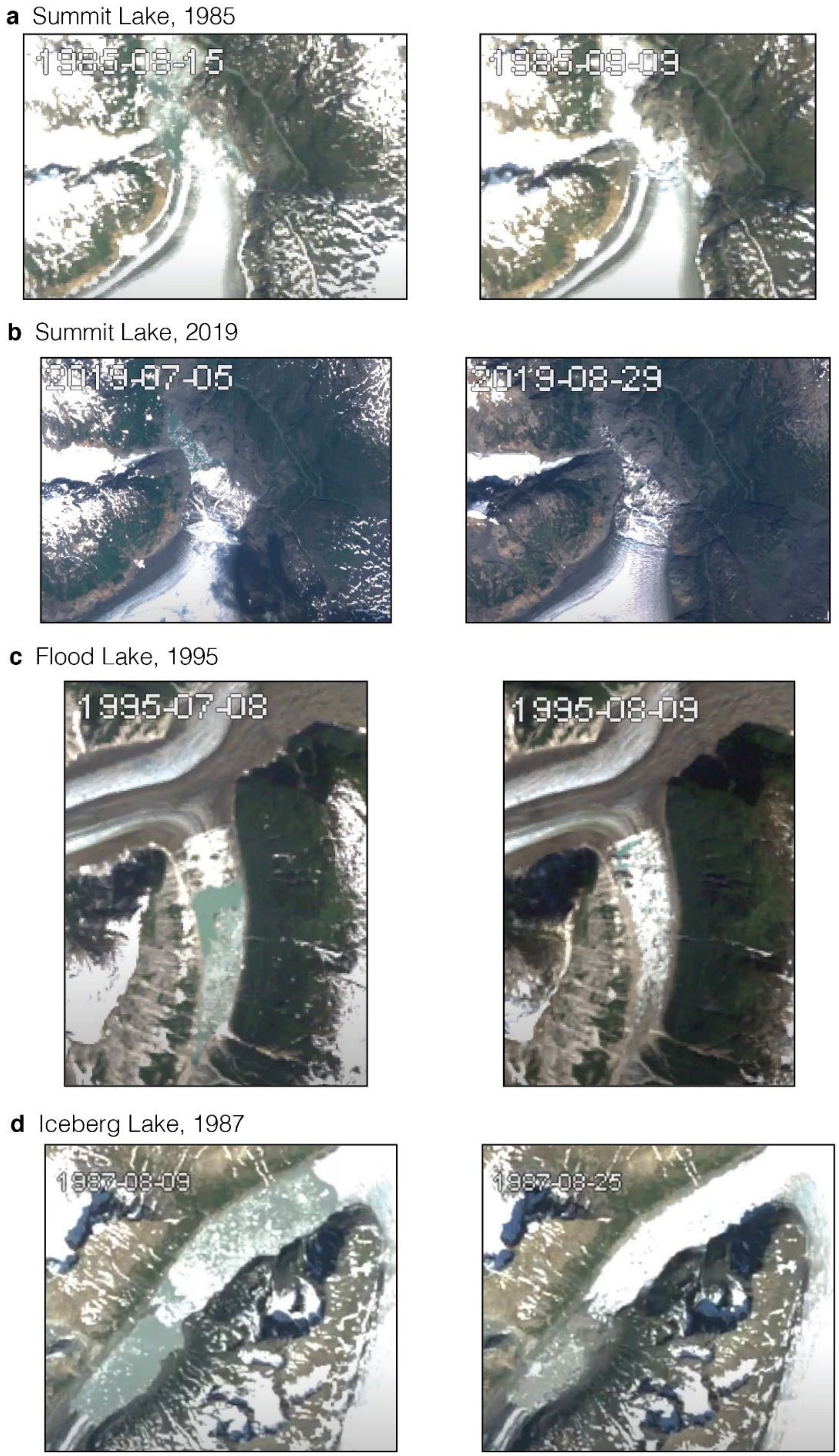

Fig. S7 | Examples of images before and after a drainage event. Example of what lakes look like before (left) and after (right) a drainage event at **a, b,** Summit Lake (SummitLake), **c,** Flood Lake (RGI60-01.03813n284), and **d** Big Lake (RGI60-01.10778n248). Landsat (a, c, d) images courtesy of the U.S. Geological Survey and Sentinel-2 (b) images courtesy of Copernicus Sentinel data.

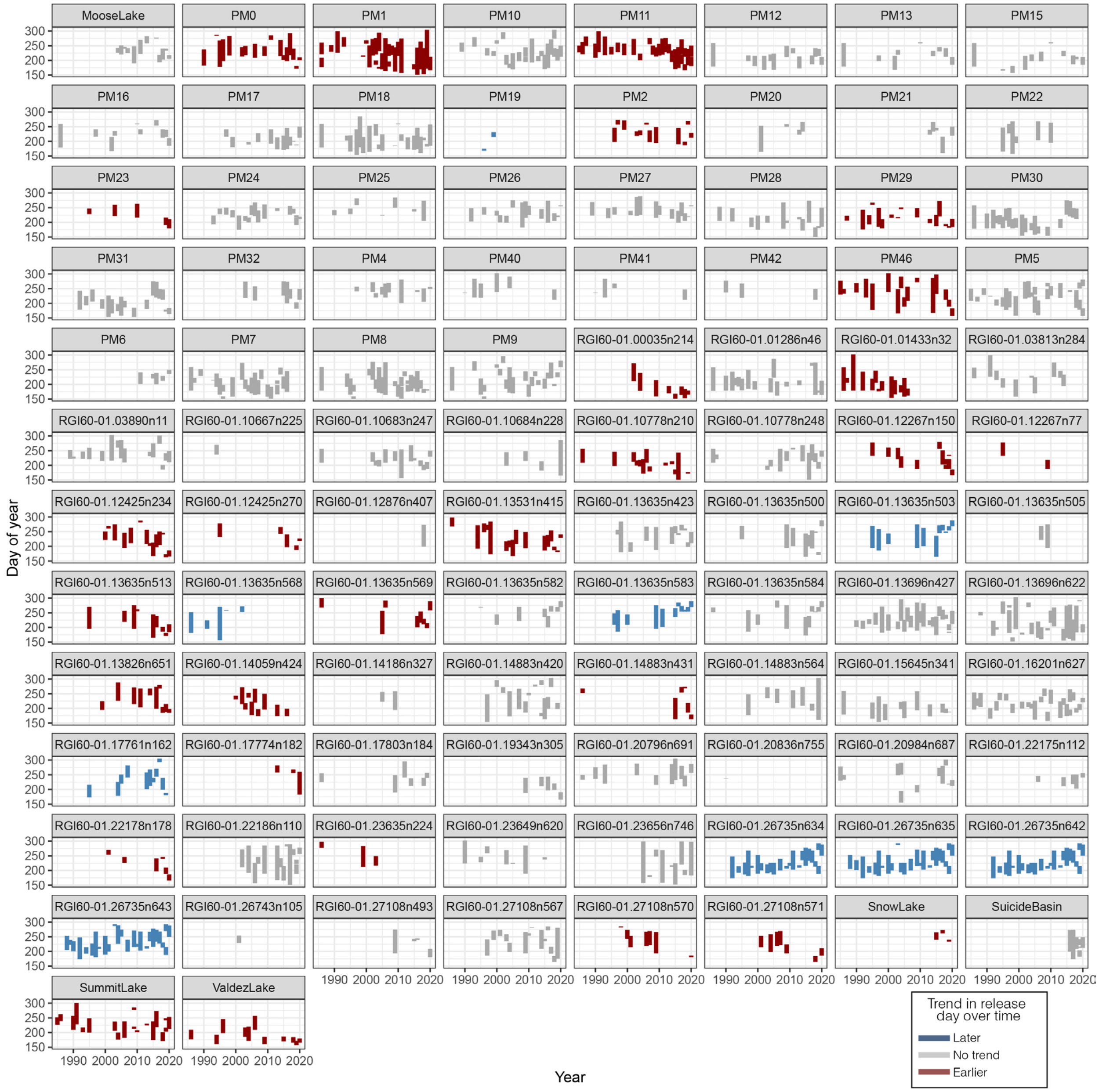

Fig. S8 | Lake drainage events documented for each individual lake. Segments signify the last date a lake was full to the first day it was drained, colored by whether the lake had an earlier release over time (red), later release over time (blue), or and indistinguishable trend (gray).

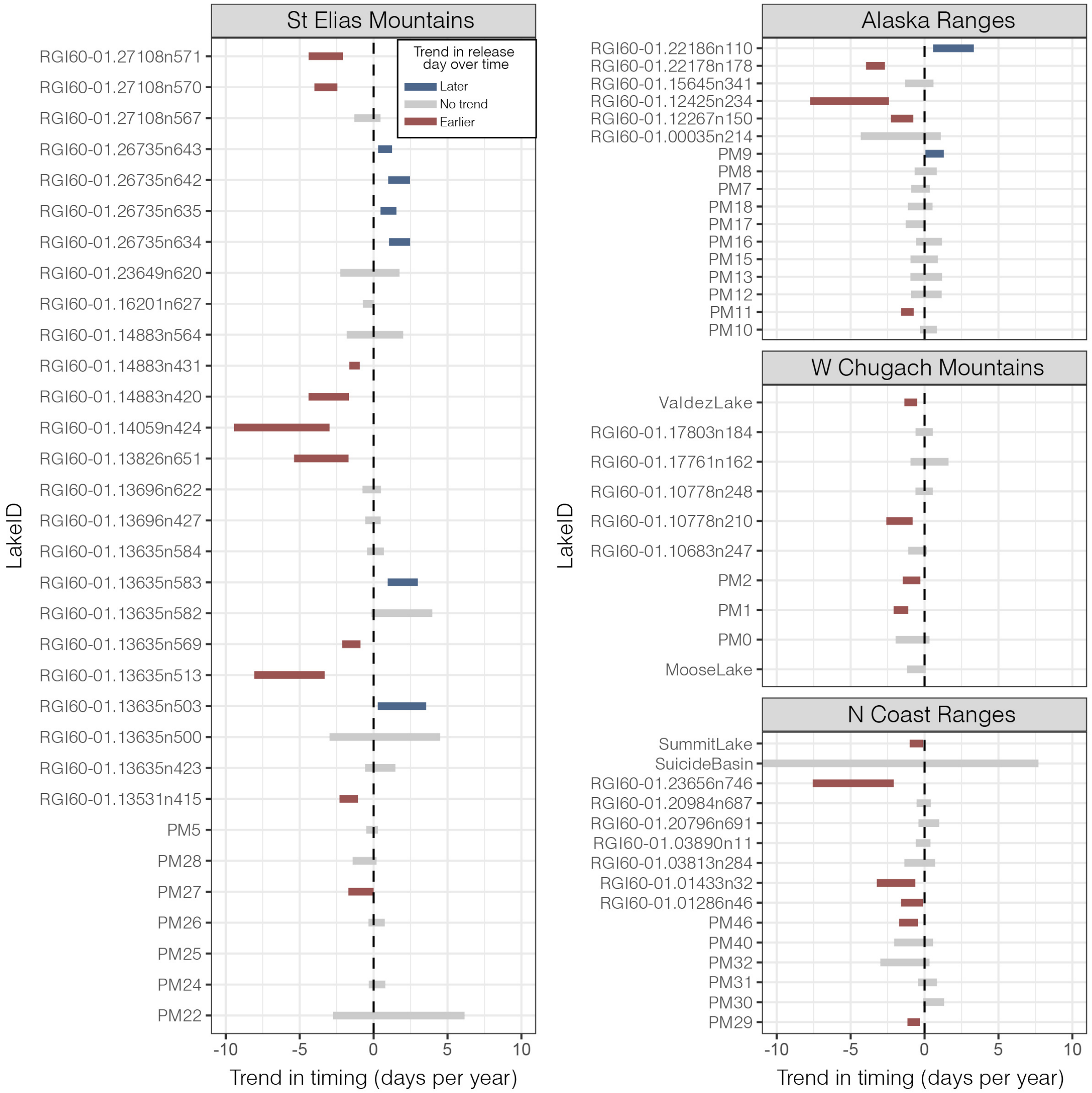

Fig. S9 | Trend in timing of lake drainage events for each individual lake. Ninety-five percent confidence interval for the trend in drainage event timing (in days per year) for each lake which experienced five or more drainage events, separated by RGI subregion (**a-d**) and colored by whether the trend indicates an earlier (red), later (blue), or undeterminable (gray) release over time.

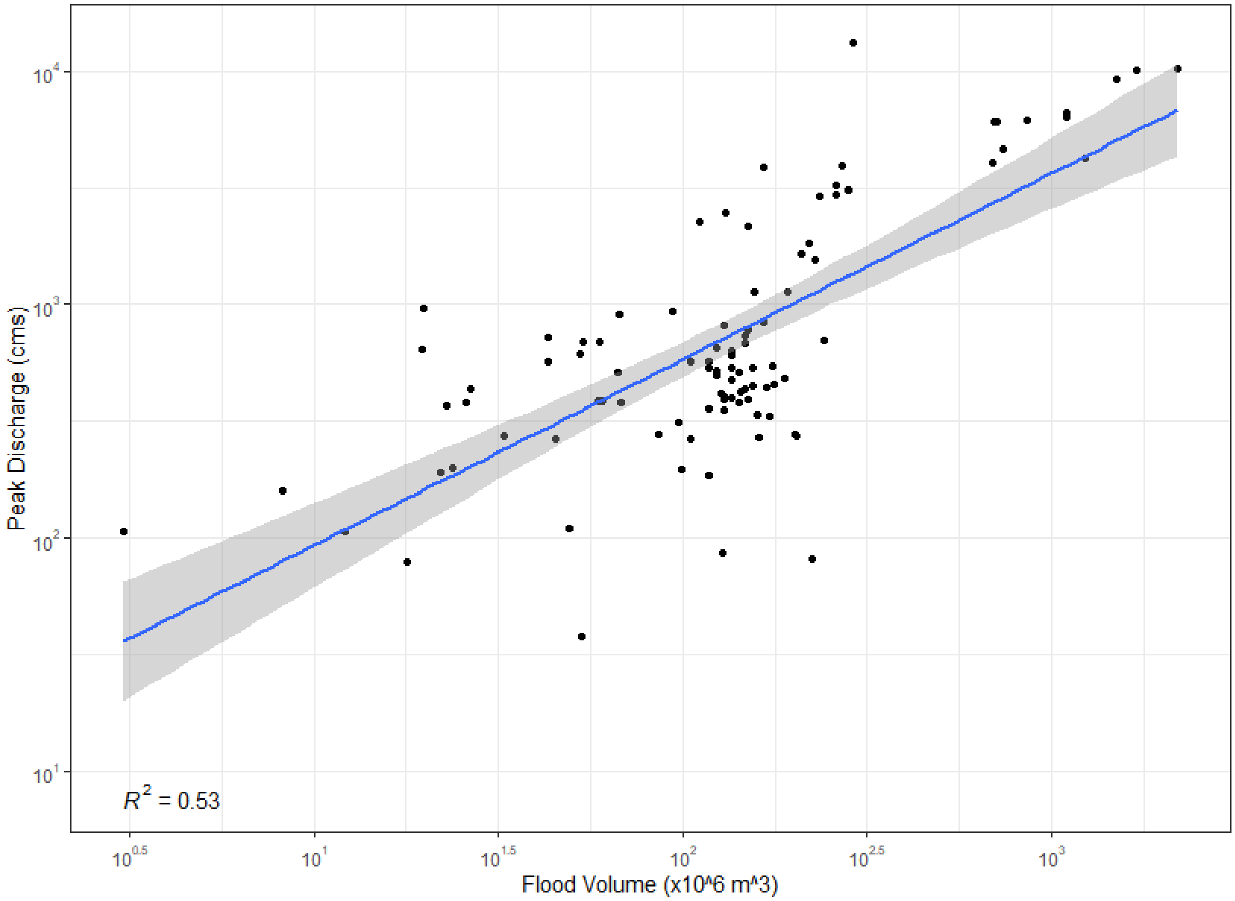

Fig. S10 | Regional relationship between flood volume and peak discharge. Black dots represent individual ice-dammed lake drainage events in Alaska with recorded flood volume and peak discharge from https://www.weather.gov/aprfc/gdlMain. Blue line represents power-law relationship with gray bars indicating the 95% confidence interval.

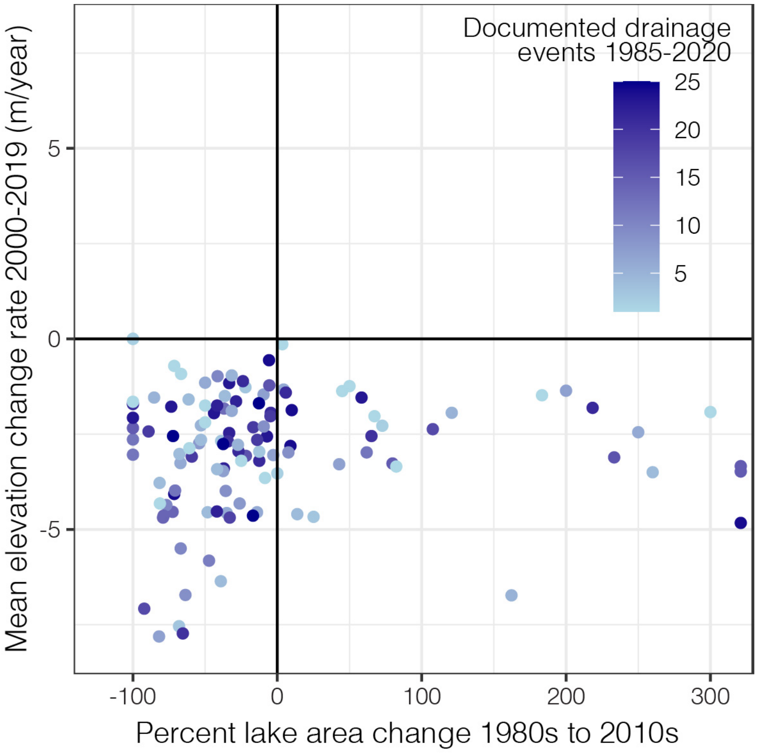

Fig. S11 | Relationship between percent lake area change and mean elevation change rate. Each dot represents an individual lake, colored by the number of documented drainage events between 1985 and 2020. Mean elevation change rate 2000-2019 is from Hugonnet et al.[3].

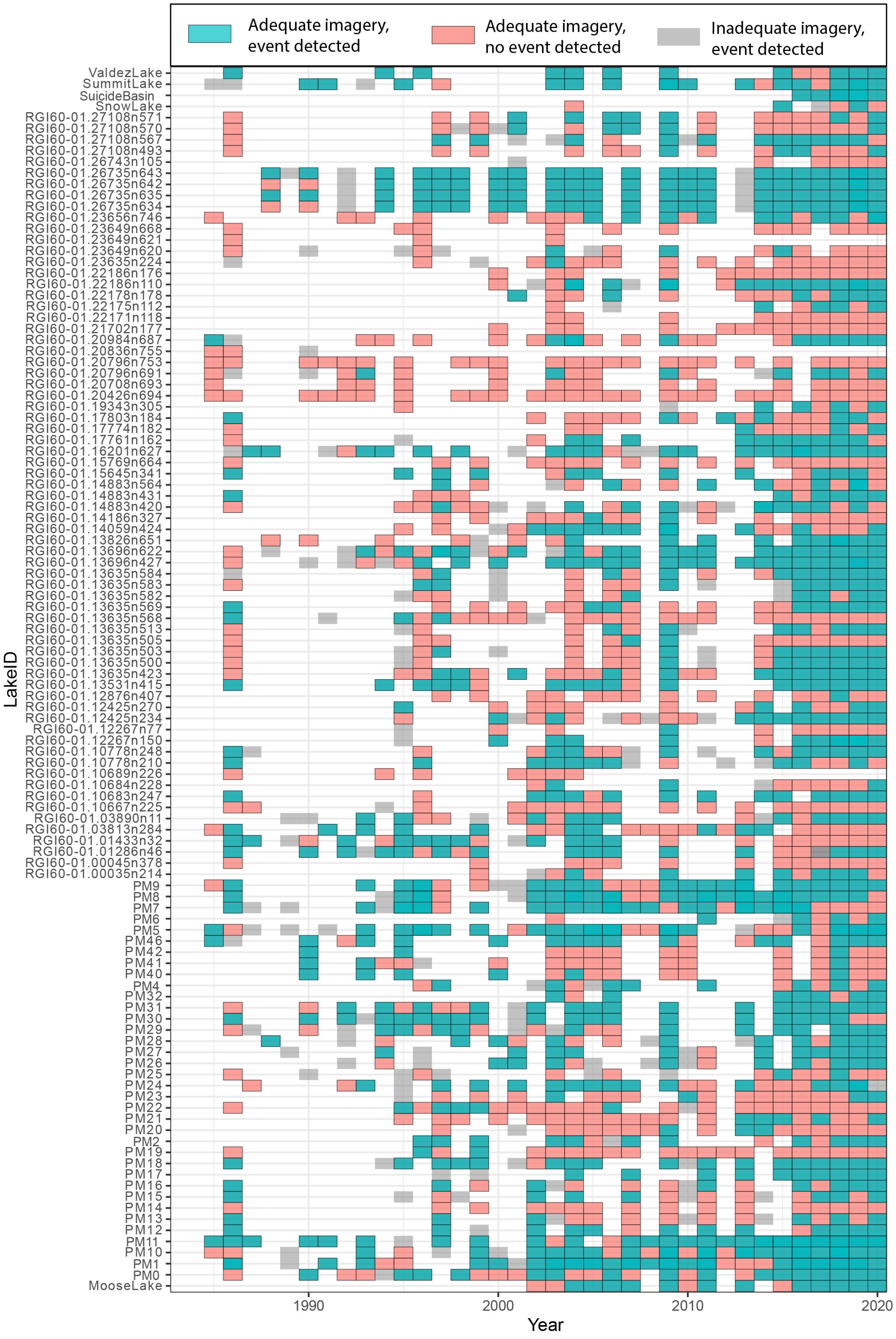

Fig. S12 | Imagery availability and drainage event detection. Lakes and years in which there was adequate imagery and an event detected (blue), adequate imagery and no event detected (red), and inadequate imagery yet an event detected (gray) given the middle ground criteria of two images at least 32 or more days apart (Fig. S3). White space represents years without adequate imagery and no events detected.

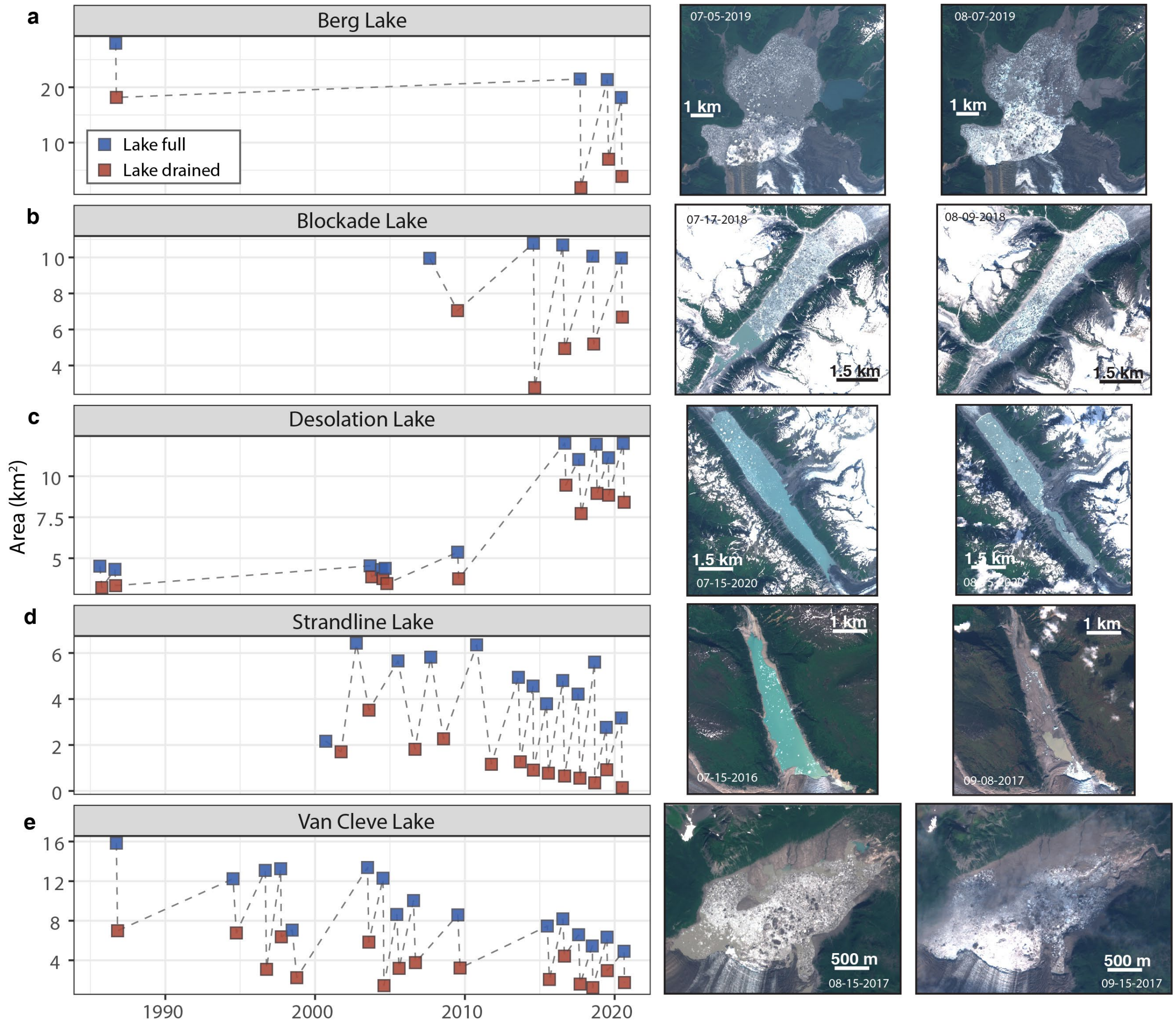

Fig. S13 | Lake area before and after a drainage event for the five largest ice-dammed lakes. a, Berg Lake (RGI60-01.14883n431), **b,** Blockade Lake (RGI60-01.19343n305), **c,** Desolation Lake (RGI60-01.20984n687), **d,** Strandline Lake (RGI60-01.12425n234), and **e,** Van Cleve Lake (RGI60-01.13531n415) and examples of Sentinel-2 imagery (Copernicus Sentinel data) before and after an event.

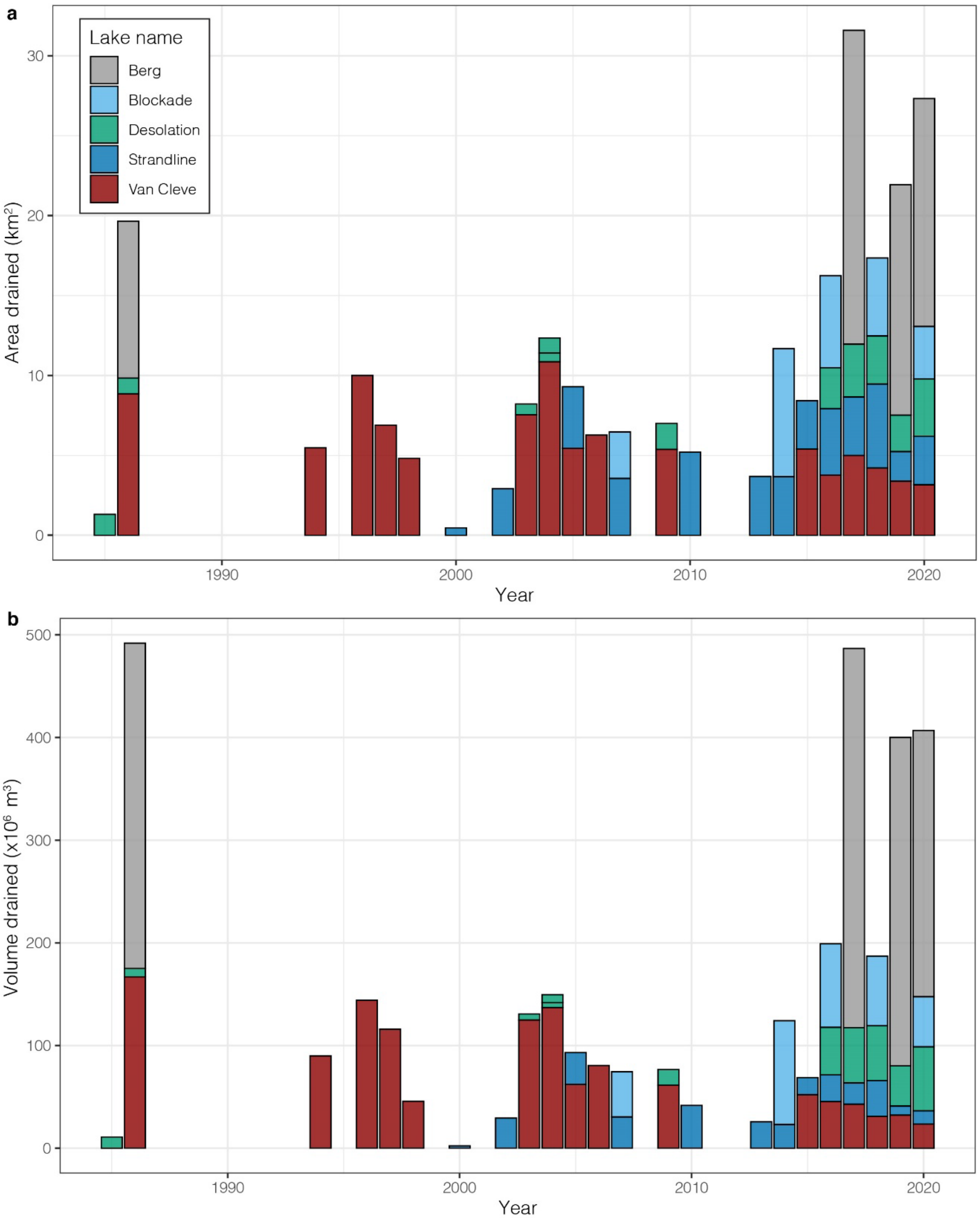

Fig. S14 | Estimated area and volume for drainage events from the five largest lakes. a, Total area and **b,** volume drained per year from the five largest ice-dammed lakes (see Fig. S6).

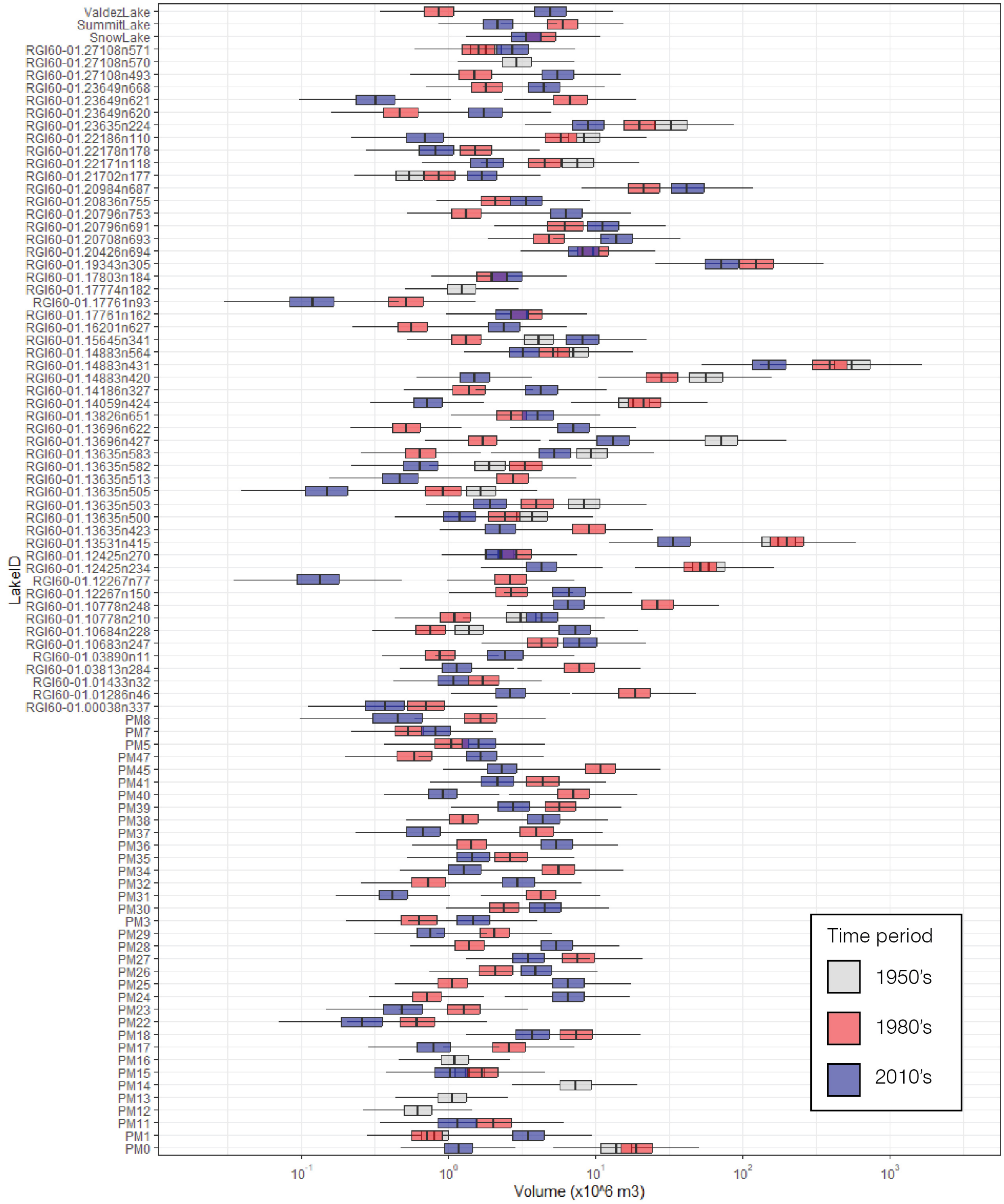

Fig. S15 | Estimated volume of lakes for the three time periods. Boxplots of the range of volumes calculated from lake area for the 1950s (gray), 1980s (red), and 2010s (blue). The box encompasses data between the 25th and 75th percentile and the line represents the median volume.

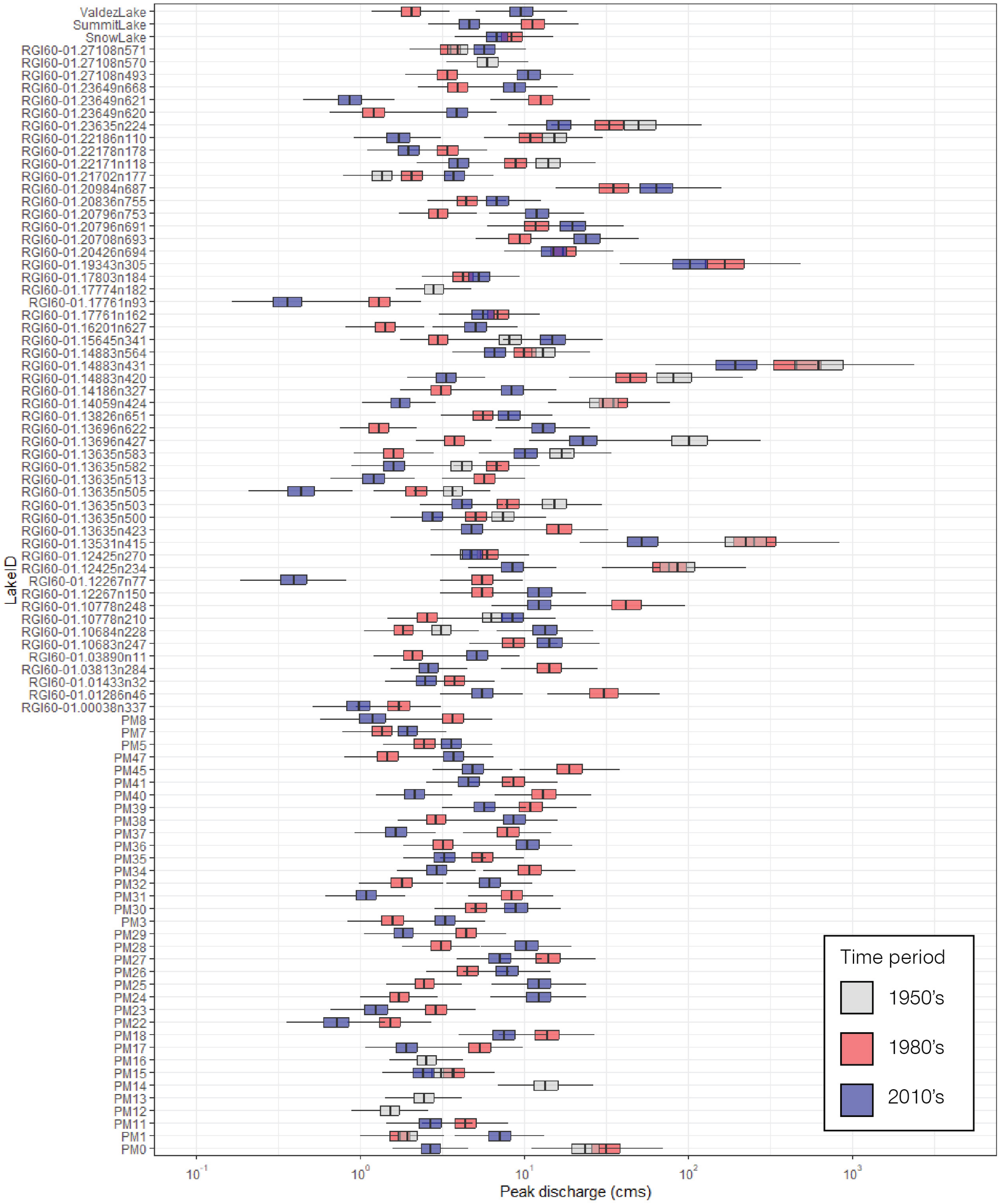

Fig. S16 | Estimated peak discharge of lakes for the three time periods. Boxplots of the range of peak discharge calculated from lake volume for the 1950s (gray), 1980s (red), and 2010s (blue). The box encompasses data between the 25th and 75th percentile and the line represents the median discharge.

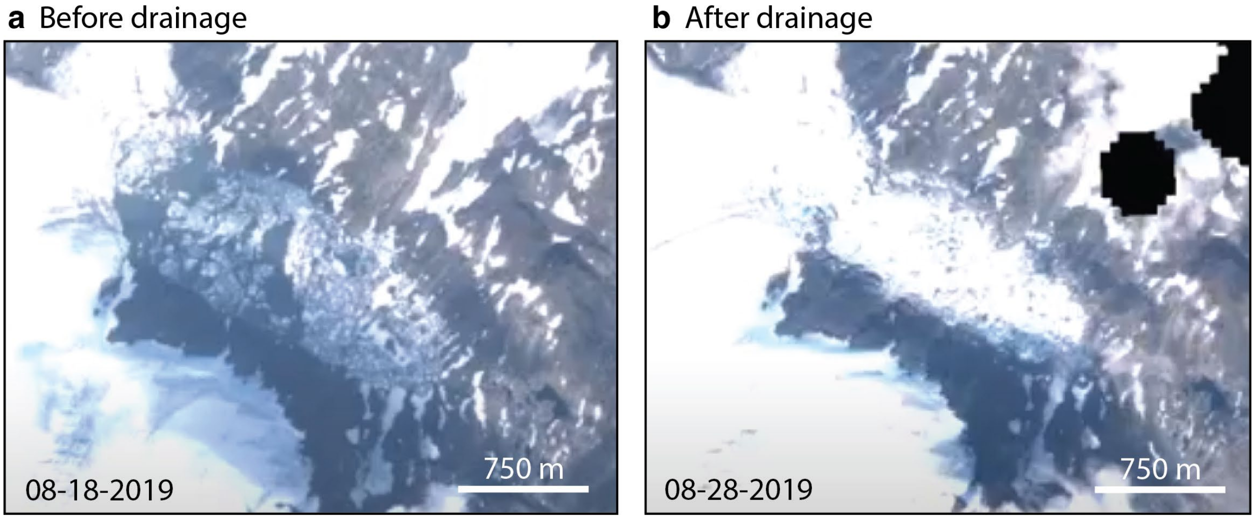

Fig. S17 | Example of a difficult to detect Snow Lake drainage event. Example of **a,** before and **b,** after a visible drainage event at Snow Lake in 2019. Sentinel-2 images (Copernicus Sentinel data) were taken ten days apart. This event resulted in $350,000 in damages[2].

References

1. Veh, G. *et al.* Trends, breaks, and biases in the frequency of reported glacier lake outburst floods. *Earth's Future* (2022) doi:10.1029/2021ef002426.

2. NOAA. Alaska GDL Main Page. *Alaska GDL Main Page* https://www.weather.gov/aprfc/gdlMain.

3. Hugonnet, R. *et al.* Accelerated global glacier mass loss in the early twenty-first century. *Nature* **592**, 726–731 (2021).
